# *Mib2* Deficiency Inhibits Microglial Activation and Alleviates Ischemia-Induced Brain Injury

**DOI:** 10.14336/AD.2019.0807

**Published:** 2020-08-07

**Authors:** Xiaoheng Li, Yajin Liao, Yuan Dong, Shuoshuo Li, Fengchao Wang, Rong Wu, Zengqiang Yuan, Jinbo Cheng

**Affiliations:** ^1^Beijing Institute for Brain Disorders, Capital Medical University, Beijing, China.; ^2^The Brain Science Center, Beijing Institute of Basic Medical Sciences, Beijing, China.; ^3^Center on Translational Neuroscience, College of Life & Environmental Science, Minzu University of China, Beijing, China.; ^4^Department of Biochemistry, Medical College, Qingdao University, Qingdao, Shandong, China.; ^5^The State Key Laboratory of Brain and Cognitive Sciences, Institute of Biophysics, Chinese Academy of Sciences, Beijing, China.; ^6^National Institute of Biological Sciences, Beijing, China.

**Keywords:** Mib2, microglia, neuroinflammation, ischemia, brain injury

## Abstract

Neuroinflammation plays a critical role in ischemia-induced brain injury. Mib2, an E3 ubiquitin ligase, has been reported to regulate Notch signaling and participate in the peripheral immune system. However, the roles of Mib2 in the nervous system are not well characterized. In this study, we show that Mib2 is involved in lipopolysaccharide (LPS)- and oxygen-glucose deprivation (OGD)-induced microglial activation. Mechanistically, Mib2 interacts with the IKK complex and regulates the activation of NF-κB signaling, thus modulating Notch1 transcription in the microglia. Furthermore, we generated a microglia-specific *Mib2* knockout mice and found that microglia-specific deletion of *Mib2 *significantly alleviates ischemia-induced neuroinflammation and brain injury. Taken together, our results reveal a critical role of Mib2 in microglial activation and ischemia-induced brain injury, thus providing a potential target for the treatment of stroke.

Neuroinflammation, generally regarded as a detrimental factor for neurological functions, is involved in various central nervous system (CNS) diseases. It can be triggered by a variety of stimuli including microbes, brain injury, toxic metabolites, autoimmune response, and neuro-degeneration [1-3]. Emerging evidence showed that neuroinflammation is tightly linked to the patho-physiology of stroke, which is the second-largest cause of death worldwide [4, 5]. During stroke, ischemia and hypoxia trigger a robust immune response and the inflammation is maintained throughout the period, from early damage to post-stroke tissue repair [4-6].

As the resident immune cell, microglia are highly ramified and motile and serve as the guardian of the CNS [2, 7]. Each microglial cell possesses its territory and constantly surveys the local microenvironment through its delicate processes [7, 8]. Upon ischemia, microglial cells, which are the first activated cells in the brain parenchyma, migrate to the infarction area and produce pro-inflammatory cytokines to initiate an immune response, thus play a critical role in stroke [9, 10]. In addition, microglial cells undergo a morphology change, from ramified to amoeboid state under which the cells display round soma and no processes [11]. Recent studies have shown that NF-κB- and Notch-signaling contribute towards the activation of microglia after stroke [12-14]. However, the precise mechanism of microglial activation in this process is not well understood.

Mind bomb-2 (Mib2) is an E3 ubiquitin ligase, which operates in conjunction with E1 ubiquitin-activating enzyme and E2 ubiquitin-conjugating enzyme, and ubiquitinates specific proteins [15]. It has been reported that Mib2 could ubiquitinate the Notch ligand to regulate the Notch signaling [16, 17]. Mib2 also participates in the regulation of NF-κB signaling and type I interferon responses [18, 19]. *Mib2* knockout mice displayed defects in the hippocampal spatial memory and contextual fear memory [20], indicating that Mib2 plays a role in the brain functions. Furthermore, the N-methyl-D-aspartate receptor NR2B subunit [21] and GABAB receptors [22, 23] have been reported as Mib2 substrates. Intriguingly, Mib2 is highly expressed in microglia [24], however, its function in microglia remains to be elucidated.

Here, we demonstrate that Mib2 promotes microglial activation by regulating NF-κB and Notch1 signaling pathways. Furthermore, microglia specific deletion of *Mib2* reduces its activation and neuroinflammation as well as brain damage after ischemic stroke, implicating that Mib2 might be a potential therapeutic target in stroke treatment.

## MATERIALS AND METHODS

### Reagents and antibodies

The following reagents were purchased: lipopoly-saccharide (LPS; Sigma-Aldrich, St. Louis, MO, USA), Tamoxifen (#S1238, Selleckchem, Houston, TX, USA). Antibodies used for immunoblotting were as follows: anti-Mib2 (#118K4777, Sigma-Aldrich), anti-iNOS/NOS Type II (#610332, BD Biosciences, San Jose, CA, USA), anti-Phospho-IKKα/β (S176/180) (16A6) (#2697P), Notch1(D1E11) (#3608S), anti-p44/42 MAPK (Erk1/2) (#9102), anti-Phospho-p44/42 MAPK (Erk1/2) (Thr202/ Tyr204) (#9101) were purchased from Cell Signaling Technology (Beverly, MA, USA), anti-IKKα (CHUK) (#A2062), anti-IKKβ (#A2087) were from ABclonal Technology (Wuhan, HB, China), anti-mouse/human CD11b (#101217), anti-mouse CD45 (#103110) were purchased from BioLegend (San Diego, CA, USA), anti-Myc (#M047-3), anti-HA (#M180-3) were from MBL (Woburn, MA, USA), anti-Flag (#F3165, Sigma-Aldrich), anti-β-tubulin (#CW0098A) and anti-GAPDH (#CW0266A) were from CWBiotech (Beijing, China), anti-β-actin (60008-1-Ig, Proteintech Group, Campbell Park, Chicago, IL, USA).

### Mice

*Mib2* conditional knockout mice were generated using the CRISPR/Cas 9 technology. Briefly, *loxP* elements were inserted upstream and downstream of exon 5 of *Mib2*, and *Mib2^flox/+^* mice were confirmed by Southern blot. To generate microglia-specific and inducible knockout mice, homozygous *Mib2^flox/flox^* mice were crossed with the mice expressing tamoxifen (TAM)-inducible Cre-recombinase under the control of the Cx3cr1 promoter (*Cx3cr1*^CreER^ mice) [25]. Mice were given TAM by intragastric administration at the age of P30 to induce microglial specific knockout. All mice were housed under a 12 h/12 h light-dark cycle at 22-24 °C with free access to water. All the animal experimental procedures were reviewed and approved by the Institutional Animal Care and Use Committee of the Beijing Institute of Basic Medical Sciences (Beijing, China).

### Cell culture and transfection

HEK 293T and BV2 cell lines were obtained from ATCC (Manassas, VA, USA) and maintained in Dulbecco's modified Eagle's medium (DMEM, Invitrogen, Waltham, MA, USA) supplemented with 10% fetal bovine serum (FBS, Gibco, Grand Island, NY, USA) and 1% Penicillin-Streptomycin (Invitrogen). All cells were maintained in a 5% CO_2_ atmosphere at 37 °C.

Transient knockdown in BV2 cells was performed by siRNA transfection with Lipofectamine^®^ RNAiMAX Transfection Reagent (#13778150, Invitrogen). The targeting sequences of siRNAs were: Mib2 1#: CUCUA UGACAACGCCCAAATT; Mib2 2#: CGCUAUGAGA CAUCUCACUTT; Notch: CCUUUCUACCGCUGUCU AUTT.

For stable knockdown in BV2 cells, shRNA against Mib2 (targeting sequence: TCGAAGGATGAAGAAGT GTAT) was used. Briefly, the complementary sequences of shRNA were annealed together and ligated into a pLKO.1 lentiviral vector (Addgene, Cambridge, MA, USA) and then co-transfected with viral packaging plasmids (VSVG and ΔR812) into HEK 293T cells. After transfection, the viral supernatant was collected (once a day for two days), centrifuged, and filtered through a 0.45 μm filter. The supernatant was used to infect the BV2 cells with polybrene (#sc-134220, Santa Cruz Biotechnology, 1:1000). Three days after the infection, cells were subjected to antibiotic selection.

### Immunoprecipitation and immunoblotting

Western blot analyses were conducted as previously described [26, 27]. For immunoprecipitation, 293T cells were lysed on ice using immunoprecipitation lysis buffer (50 mM Tris-HCl (pH 7.4), 150 mM NaCl, 1 mM EDTA, 1 mM EGTA, 5 mM Na4Ppi, 25 mM NaF, 1% Triton X-100, 1 mM PMSF, and protease inhibitor cocktail) for 20 min, followed by centrifugation at 13000 rpm for 15 min. The supernatant was pre-cleared with protein G agarose beads followed by immunoprecipitation at 4 °C for 2 h using IgG or specific antibody-conjugated beads. The immunoprecipitates were washed five times with the immunoprecipitation wash buffer (50 mM Tris-HCl (pH 7.4), 150 mM NaCl, 1 mM EDTA, 1 mM EGTA, 5 mM Na4Ppi, 25 mM NaF, 0.5% Triton X-100) and centrifuged at 3000 rpm for 3 min each time. The immunoprecipitated proteins were eluted using SDS-PAGE loading buffer and analyzed by western blot.

### The IKK complex ubiquitylation assays

The ubiquitination assays were performed as previously described [28]. The cells were lysed on ice in lysis buffer (50 mM Hepes (pH 7.4), 150 mM NaCl, 1% Nonidet P-40, 0.1% deoxycholate, 0.05% SDS, 0.1 M NaF, 1 mM EGTA and protease inhibitor cocktail) for 20 min, followed by centrifugation at 14000 rpm for 15 min. The supernatant was pre-cleared and then incubated with Myc antibody-conjugated beads for 3 h. Then the beads were washed 4 times using lysis buffer, and the proteins were eluted using SDS-PAGE loading buffer and analyzed by western blot.

### Dual-luciferase reporter system

Mouse NF-κB and Hes1 reporters were generated in our laboratory. Notch1 promoter (-2000 bp to +550?bp) was cloned into a pGL3-luciferase reporter vector (Promega, Madison, WI, USA). The control pCMV-Renilla plasmid and the other plasmids were co-transfected into 293T cells. 16 h after transfection, the cells were lysed, and the luciferase activity was measured.

### Isolation of murine microglia and flow cytometry analysis

Microglia were isolated by Percoll density gradient purification [29, 30]. In brief, fresh brain tissue was rinsed twice with PBS with 2% FBS, disrupted mechanically and then enzymatically digested using type IV collagenase (#17104019, Gibco) for 15 min at 37 °C. The suspension was filtered through a 70 μm filter and centrifuged at 600 g for 6 min at 4 °C. The pellet was resuspended in 37% Percoll (diluted by PBS) and added to a graduated Percoll (#17-0891-01, GE Healthcare, Uppsala, Sweden) gradient prepared in a 15 mL conical bottomed tube followed by centrifugation at 2000 g for 20 min at 4 °C. The cell layer was collected and rinsed with PBS and centrifuged at 600 g for 6 min at 4 °C. After centrifugation, the supernatant was discarded, and the cells were resuspended in PBS (with 2% FBS) and stained with anti-CD11b-FITC and anti-CD45-Cy5 (Biolegend) antibodies at concentrations recommended by the manufacturer and incubated at room temperature in dark for 30 min. The stained cells were analyzed and sorted on the FACS Vantage (BD Biosciences) flow cytometer. The sorted microglial cells were collected by centrifugation and lysed using TRIzol^TM^ Reagent (#15596018, Invitrogen) for RNA isolation and subsequent RT-qPCR.

### RNA isolation and RT-qPCR

Tissues, cultured cells, and sorted cells were lysed in TRIzol^TM^ Reagent. Tissues were finely chopped in TRIzol and immediately homogenized using a homogenizer. Total RNA was isolated according to the manufacturer’s instructions. mRNA was converted to cDNA using the cDNA synthesis kit (AE311-03, TransGen Biotech, Beijing, China). RT-qPCR was performed with the diluted cDNA in three wells for each primer using SYBR green master mix (Bio-Rad) on a Bio-Rad iCycler iQ Real Time PCR system. All RT-qPCR experiments were repeated at least three times. The primers for RT-qPCR are shown in Supplementary Table 1.

### Immunofluorescence

Tissues were formalin-fixed, paraffin-embedded, and sectioned. After de-paraffinization and rehydration, antigen retrieval was performed in citrate buffer (pH 6.0) for 20 min at 95-100 °C. The slides were then treated with 10% normal horse serum for 1 h at room temperature followed by incubation with anti-Iba1 antibody (1:400 at 4 °C overnight, Wako, 019-19741, Chuo-Ku, Osaka, Japan). The slides were then washed and incubated with the fluorescence conjugated secondary antibody in dark for 1 h at room temperature. After, the sections were rinsed and incubated with DAPI (Invitrogen) and visualized by immunofluorescent microscopy. The staining of all the slides was blinded. The number of Iba1 positive cells and soma area were analyzed at 20× magnification using Image J software (NIH, Bethesda, MD, USA).

### TTC Staining

After 24 h or 72 h of reperfusion, mice were anesthetized and the brain was perfused using saline. After, the brain was cut into eight 1 mm serial coronal sections and incubated in 1.5% 2,3,5-triphenyltetrazolium chloride (TTC; *v*/*v *in saline, Sigma-Aldrich) solution for 5 min at 37 °C as previously described [31-33]. Images were obtained and the infarct volumes were analyzed using ImageJ software. The final infarct volumes were presented as a ratio of the volume of ischemic tissue area to the total volume in all serial coronal sections.

### tMCAo model

For focal cerebral ischemia, we induced right-side transient tMCAo as described previously [33]. For reperfusion, we removed the monofilament from the MCA and the suture occluding CCA (common carotid artery) 75 min after the occlusion.

### OGD model

For oxygen-glucose deprivation, cells were rinsed and incubated in glucose-free Minimum Essential Medium (MEM; Gibco) and then transferred to an anaerobic chamber containing a mixture of 95% N2, and 5% CO_2_ and incubated at 37 ? for 3 h. Cells were then switched to the normal culture medium and culture conditions to terminate the OGD treatment.

### Statistical analysis

The results are shown as mean ± SEM. Statistical analysis was performed using ImageJ software and GraphPad Prism 6 (GraphPad Software, San Diego, CA, USA). T-test was used to compare two groups and one-way ANOVA for multiple groups. *p* < 0.05 was considered statistically significant.

**Figure 1.**
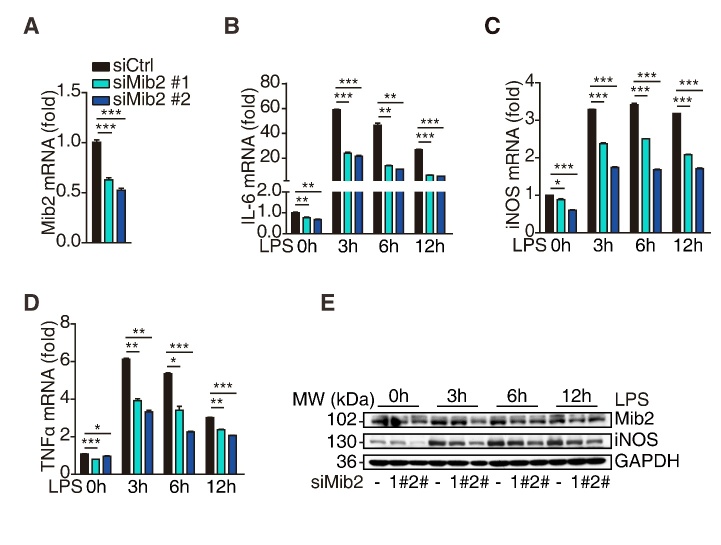
**Mib2 knockdown inhibits LPS-induced inflammation**. (**A**), The knockdown efficiency was determined by RT-qPCR analysis after transfected with Mib2 or control siRNA in BV2 cells for 72h. (**B-D**), The expression levels of IL-6, iNOS and TNFα in control and Mib2 knockdown-BV2 cells were analyzed upon LPS (1 µg/ml) stimulation for indicated times. (**E**), Western blot analysis of Mib2 and iNOS levels in control and Mib2 knockdown-BV2 cells upon LPS (1 µg/ml) stimulation for indicated times. Data indicate means ± SEM. Data were analyzed using one-way ANOVA. *p < 0.05, **p < 0.01, ***p < 0.001.

## RESULTS

### Mib2 promotes microglial activation in vitro

To investigate whether Mib2 is involved in the microglial activation, we stimulated BV2 microglial cell line with lipopolysaccharide (LPS, 1μg/mL) for different time points. We found that the mRNA levels of Mib2 were significantly increased after LPS stimulation (Supplementary Fig. 1A), indicating that Mib2 might be involved in LPS-induced microglial activation. Previous studies showed that ischemic stroke could strongly trigger microglial activation and neuroinflammation [34, 35]. In the oxygen and glucose deprivation (OGD) model, an *in vitro* model of ischemia-induced damage, we observed that the mRNA levels of Mib2 were significantly upregulated after 3 h of OGD followed by 6 h of re-oxygenation (Supplementary Fig. 1B). Similarly, in the animal model of transient middle cerebral artery occlusion (tMCAo), Mib2 levels were significantly increased after 3 h and 9 h of reperfusion (Supplementary Fig. 1C), suggesting that Mib2 might be involved in the early stages of stroke. Taken together, these results indicate that Mib2 is involved in the microglial activation.

**Figure 2.**
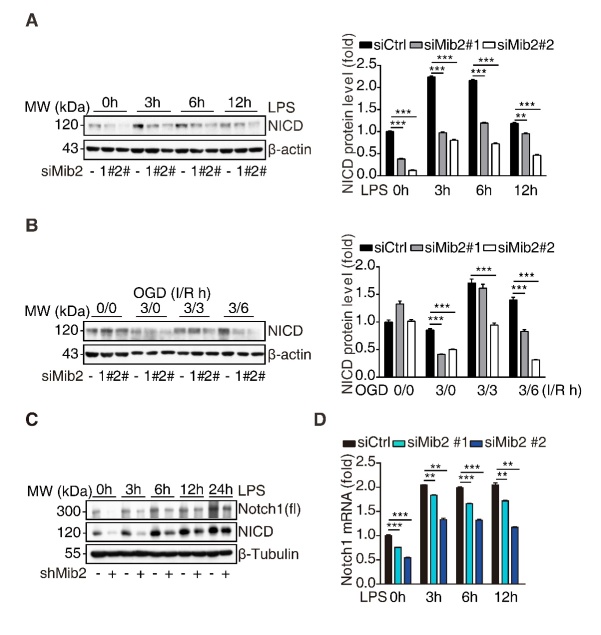
**Mib2 regulates Notch1 signaling in microglia**. (**A**) Western blot analysis of NICD levels (left) and relative band density quantification (right) in control and Mib2 knockdown-BV2 cells upon LPS (1 µg/ml) stimulation for indicated times. (**B**) Western blot analysis of NICD levels (left) and relative band density quantification (right) in control and Mib2 knockdown-BV2 cells upon OGD treatment (ischemia for 3h and reperfusion for indicated times), I: ischemia, R: reperfusion. (**C**) Western blot analysis of Notch1 (fl, full length) and NICD levels in control and Mib2 knockdown-BV2 cells upon LPS (1 µg/ml) stimulation for indicated times. (**D**) RT-qPCR analysis of Notch1 expression levels in control and Mib2 knockdown-BV2 cells upon LPS (1 µg/ml) stimulation for indicated times. Data indicate means ± SEM. Data were analyzed using one-way ANOVA. *p < 0.05, **p < 0.01, ***p < 0.001.

To explore the role of Mib2 in microglial activation, we knocked down Mib2 in BV2 cells (Fig. 1A) and treated the cells with LPS (1μg/mL) for different time points. As shown in Fig. 1B-1D, LPS stimulation significantly increased the levels of pro-inflammatory markers, including IL-6, iNOS, and TNFα, which are reported to exacerbate brain damage during stroke [35]. Importantly, Mib2 knockdown inhibited the upregulation of these cytokines upon LPS stimulation. Further, the protein levels of iNOS were also decreased in Mib2 knockdown cells as compared to the control cells upon LPS stimulation (Fig. 1E), suggesting that Mib2 plays a critical role in microglia-mediated neuroinflammation. Similar results were obtained in the Mib2-knockdown stable BV2 cell line (Supplementary Fig. 2A-D). These data indicate that Mib2 functions as a positive regulator of microglial activation.

**Figure 3.**
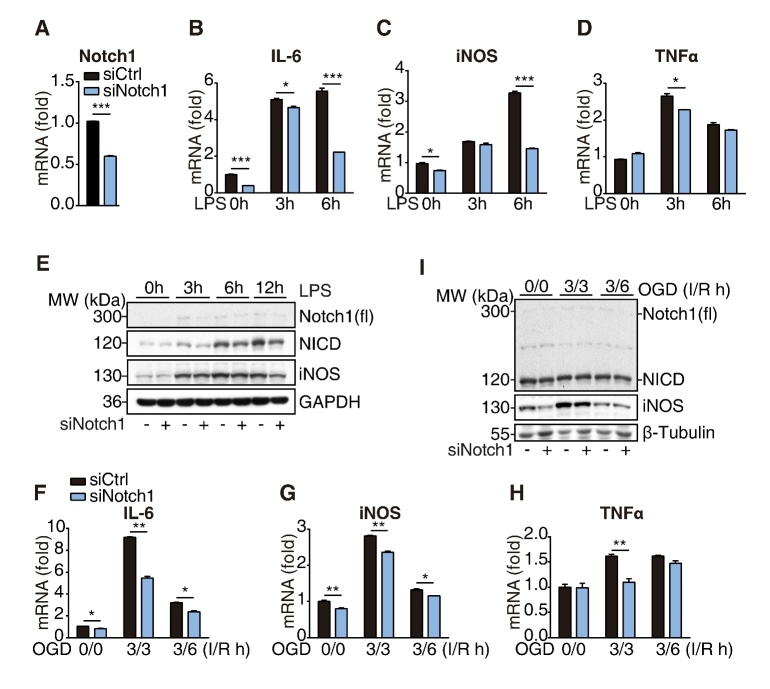
**Notch1 signaling regulates microglial activation**. (**A**) The knockdown efficiency was determined by RT-qPCR analysis after transfected with Notch1 or control siRNA in BV2 cells for 72h. (**B-D**) The expression levels of IL-6, iNOS and TNFα in control and Notch1 knockdown-BV2 cells were analyzed upon LPS (1 µg/ml) stimulation for indicated times. (**E**) Western blot analysis of indicated proteins from control and Notch1 knockdown-BV2 cells upon LPS (1 µg/ml) stimulation for indicated times. (**F-H**) The expression levels of IL-6, iNOS and TNFα in control and Notch1 knockdown-BV2 cells were analyzed upon OGD treatment (ischemia for 3h and reperfusion for indicated times), I: ischemia, R: reperfusion. (**I**) Western blot analysis of indicated proteins from control and Notch1 knockdown-BV2 cells upon OGD treatment (ischemia for 3h and reperfusion for indicated times), I: ischemia, R: reperfusion. Data indicate means ± SEM. Data were analyzed using Student’s *t* test and one-way ANOVA. *p < 0.05, **p < 0.01, ***p < 0.001.

**Figure 4.**
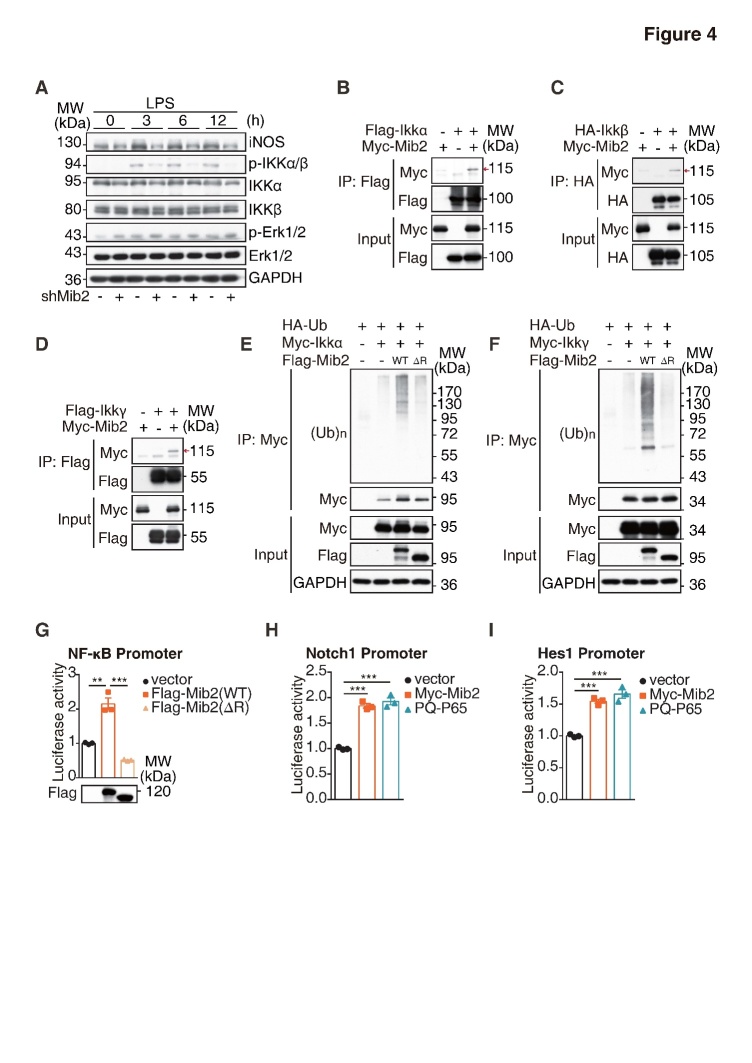
**Mib2 regulates NF-κB signaling by targeting IKK complex**. (**A**) Western blot analysis of iNOS and the phosphorylated and total IKKα, IKKβ, Erk, P38 levels upon LPS stimulation for indicated times in control and Mib2 knockdown BV2 cells. (**B**) 293T cells were transfected with Myc-tagged Mib2 and Flag-tagged IKKα. Cell lysates were immunoprecipitation with Flag antibody and co-immunoprecipitation of Myc-tagged Mib2 was detected by Western blot analysis. (**C**) 293T cells were transfected with Myc-tagged Mib2 and HA-tagged IKKβ. Cell lysates were immunoprecipitation with HA antibody and co-immunoprecipitation of Myc-tagged Mib2 was detected by Western blot. (**D**) 293T cells were transfected with Myc-tagged Mib2 and Flag-tagged IKKγ. Cell lysates were immunoprecipitation with Flag antibody and co-immunoprecipitation of Myc-tagged Mib2 was detected by Western blot analysis. (**E-F**) 293T cells were transfected with Flag-tagged Mib2 WT or ΔR (Ring domain deletion) or vector plasmid together with HA-tagged ubiquitin, Myc-tagged IKKα or IKKγ. Cell lysates were immunoprecipitated with Myc antibody and immunoblotted with HA antibody. (**G**) 293T cells were co-transfected with NF-κB luciferase reporter plasmids and Flag-Mib2 WT or ΔR (Ring domain deletion) or vector plasmid, 16 h after transfection, cells were lysed, and the fluorescence values were measured. (**H-I**) 293T cells were co-transfected with Notch1 or Hes1 luciferase reporter plasmids and Myc-Mib2, P65 or vector plasmid, 16 h after transfection, cells were lysed, and the fluorescence values were measured. Data indicate means ± SEM. Data were analyzed using Student’s *t* test. *p < 0.05, **p < 0.01, ***p < 0.001.

**Figure 5.**
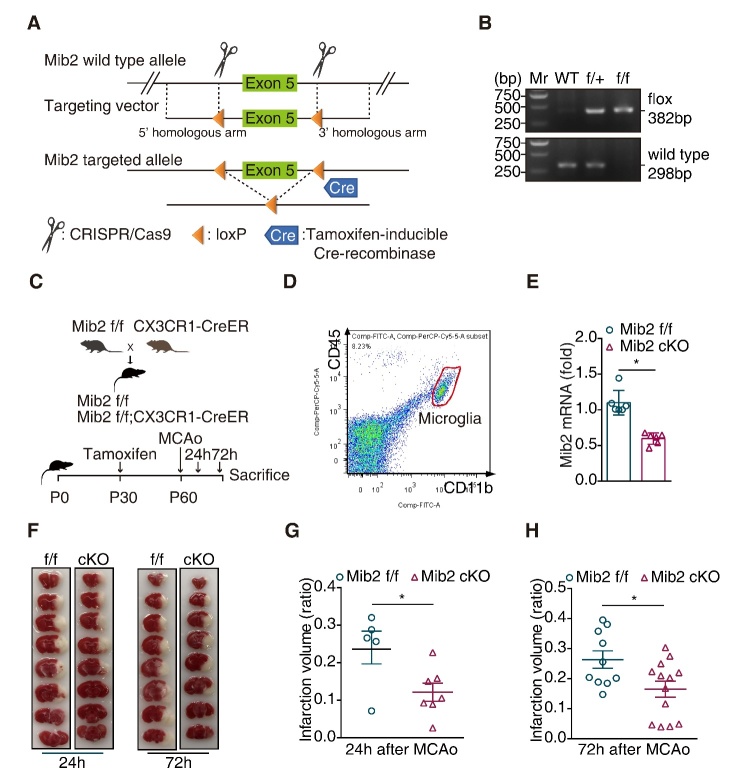
**Microglial *Mib2* knockout alleviates ischemia induced brain injury**. (**A**) Gene strategy for *Mib2^f/f^* mice and *Mib2* conditional knockout (*Mib2* cKO) mice: The *loxP* elements were inserted upstream and downstream of exon 5 by using the CRISPR/Cas 9 method. The recombination between the *loxP* sites was occurred and the target sequence was deleted when Tamoxifen-inducible Cre-recombinase was expressed. (**B**) Identification of mouse genotypes by agarose gel electrophoresis. The upper panel is for the identification of *loxP* site, the lower panel s for the identification of wild-type allele. WT for wild-type; f/+ for Mib2 heterozygous mice; f/f for Mib2 homozygous mice. (**C**) Schematic of the experimental design: *Mib2^f/f^* mice were crossed with *Cx3Cr1*-creER mice to obtain *Mib2^f/f^* and *Mib2^f/f^ Cx3Cr1*-creER littermates. These littermates were then given tamoxifen (200 μg/g by intragastric administration) for three consecutive days at one-month-old to induce Mib2*^f/f^* contrast and *Mib2* conditional knockout (*Mib2* cKO) mice. tMCAo was performed after 30-days-tamoxifen administration, mice were then sacrificed after 24 or 72 h reperfusion. (**D**) Microglia was isolated by flow cytometry from normal 2-month-old mice brain in Mib2*^f/f^* and *Mib2* cKO group (CD11b^+^CD45^low^ cells), (n = 3 mice per group). (**E**) The Mib2 knockdown efficiency was determined by RT-qPCR analysis. (**F**) Relative images of TTC stained brain slices were shown at different time points. (**G**) Brain infarction volume was quantified at 24 h after tMCAo (n=5 and 7). (**H**), Brain infarction volume was quantified at 72 h after tMCAo (n=10 and 13). Data indicate means ± SEM. Data were analyzed using Student’s *t* test. *p < 0.05, **p < 0.01, ***p < 0.001.

### Mib2 transcriptionally regulates Notch1 signaling pathway

Next, we asked how Mib2 regulates microglial activation. It has been reported that Mib2 regulates Notch signaling by functioning as an E3 ubiquitin ligase and promoting the endocytosis of Delta, a Notch signaling ligand [17]. Using the Ubibrowser prediction system, we found that the predicted substrates of Mib2 are mainly associated with the Notch signaling pathway [36]. Therefore, we asked whether Notch signaling is involved in Mib2-mediated microglial inflammation. To address this, we knocked down Mib2 in BV2 cells. Interestingly, we found that the protein levels of the Notch1 intracellular domain (NICD), an activated form of Notch1, were significantly reduced upon Mib2 knockdown. Mib2 knockdown also markedly reduced the LPS-induced elevation of NICD (Fig. 2A), which was confirmed in the Mib2-knockdown stable BV2 cell line (Fig. 2C). Similarly, in the OGD model, we observed that Mib2 knockdown significantly reduced the re-oxygenation-induced elevation of NICD (Fig. 2B and Supplementary Fig. 3A). Since Mib2 is an E3 ubiquitin ligase that putatively recruits enzyme to its substrate proteins and targets it for degradation, we would have observed an upregulation of NICD upon Mib2 knockdown if NICD is a downstream target of Mib2. However, we found that the protein levels of NICD were reduced upon Mib2 knockdown, which failed to support that NICD is a downstream target of Mib2 ubiquitination. We then asked whether Mib2 regulates the NICD transcription. As shown in Fig. 2D, we found that Mib2 knockdown decreased the levels of Notch1 transcript and prevented the LPS-induced Notch1 upregulation, which was confirmed in the Mib2-knockdown stable BV2 cell line (Fig. 2C and Supplementary Fig. 3B). Together, these results suggest that Mib2 transcriptionally regulates Notch1 signaling in microglia.

### Notch1 promotes microglial activation

Since Mib2 transcriptionally regulates the expression of Notch1/NICD in microglia, we examined the role of Notch1 signaling in microglia-mediated inflammation. We used siRNA to knockdown Notch1 in BV2 cells and treated the cells with LPS (1μg/mL) for various time points. The results show that the mRNA levels of pro-inflammatory markers, including IL-6, iNOS, and TNFα, were markedly reduced upon Notch1 knockdown (Fig. 3A-3D). Consistently, the protein levels of iNOS were also reduced in Notch1 knockdown cells (Fig. 3E), suggesting that Notch1 promotes microglia-mediated neuroinflammation. Notch1 knockdown also inhibited the re-oxygenation-induced microglial activation in the OGD model (Fig. 3F-3I), which is consistent with the results in LPS-induced inflammation. Taken together, these results demonstrate that Notch1 promotes microglia-mediated inflammation. Given that Mib2 regulates the expression levels of Notch1 in the microglia, we argued that Notch1 is involved in Mib2-mediated microglial activation.

### Mib2 participates in microglia-mediated inflammation by targeting IKK complex of NF-κB signaling

In the peripheral immune system, Mib2 has been shown as an important regulator of the NF-κB signaling [18]. We hypothesized that Mib2 regulates Notch1 expression through NF-κB signaling during LPS- and OGD-induced microglial activation. To address this, we examined the changes in the key components of NF-κB signaling. As shown in Fig. 4A, LPS stimulation increased the protein levels of p-IKKα and p-IKKβ, which were dramatically reduced upon Mib2 knockdown. Further, we found that Mib2 interacts with IKKα,IKKβ, and IKKγ (Fig. 4B-4D). Moreover, Mib2 promotes the ubiquitination levels of IKKα and IKKγ, while shows no effect on IKKβ (Fig. 4E, 4F and Supplementary Fig. 4). Meanwhile, Mib2 with RING domain deletion, which lacks the E3 ligase activity, failed to promote the ubiquitination, suggesting that Mib2 interacts and ubiquitinates the IKK complex in microglia.

We then evaluated the role of Mib2 in the activation of NF-κB signaling. Utilizing a dual-luciferase reporter system, we found that the expression of NF-κB promoter-luciferase was significantly increased upon overexpression of wild type Mib2, while it was remarkably reduced by the overexpression of RING-domain deleted Mib2 (Fig. 4G), suggesting that Mib2 mediated regulation of NF-κB signaling is dependent on its E3 ubiquitin ligase activity. Accordingly, we found that the levels of both Notch1 and its downstream target, Hes1, were markedly increased upon overexpression of Mib2 and p65, an important transcription factor in the NF-κB signaling (Fig. 4H-4I), indicating that Notch1 transcription is regulated by NF-κB signaling. Together, these results suggest that Mib2 participates in microglia-mediated inflammation through regulating NF-κB signaling.

### Microglial Mib2 knockout alleviates neurinflammation and brain injury during stroke

To explore the function of Mib2 *in vivo*, we generated the inducible *Mib2* microglia-specific knockout mice (*Mib2* cKO mice) by crossing the *Mib2^flox/flox^* mice with the mice expressing tamoxifen (TAM)-inducible Cre-recombinase under the control of the Cx3cr1 promoter (*Cx3cr1*^CreER^ mice). At postnatal day 30, mice were administered TAM and 30 days later mice were subjected to right-side tMCAo (Fig. 5A-5C). Mib2 knockout in microglia was confirmed by flow cytometry (Fig. 5D) and RT-qPCR (Fig. 5E). As shown in Fig. 5E, the levels of *Mib2* in the cKO mice were largely reduced compared to the *Mib2^flox/flox^* mice. Further, using TTC staining, we found that the infarct volumes were significantly reduced in *Mib2* cKO mice compared with the littermates of WT mice at both, 24 h- and 72 h-reperfusion (Fig. 5F-5H), suggesting that microglia specific deletion of *Mib2* alleviates ischemia-induced brain injury.

**Figure 6.**
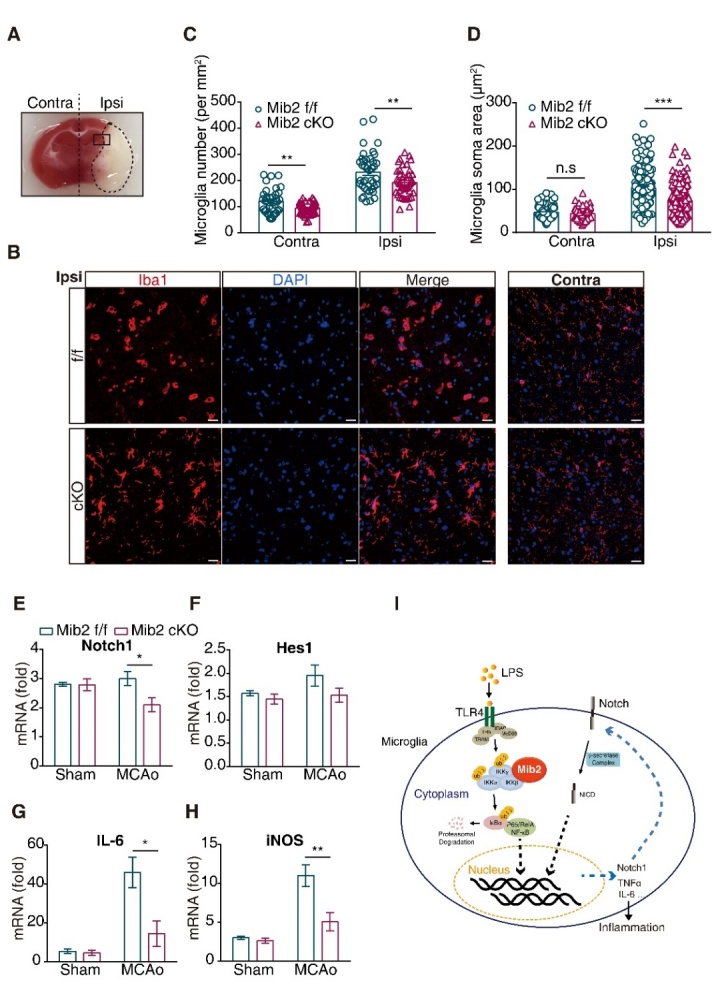
**Microglial *Mib2* knockout reduces microglial activation and inflammation**. (**A**) The contralateral and ipsilateral of brain slices from mice underwent tMCAo and the location of ischemic penumbra. (**B**) Immunofluorescence staining representation of microglia in ischemic penumbra and the corresponding position on contralateral of mice underwent tMCAo after 72h reperfusion. Scale bars, 20 μm. (**C**) Quantification of microglial numbers in ischemic penumbra and the corresponding position on contralateral. (**D**) Quantification of microglial soma area in ischemic penumbra and the corresponding position on contralateral. (**E-H**), RT-qPCR analysis of Notch1, Hes1, IL-6 and iNOS expression levels in ischemic penumbra regions of brain tissues. (**I**) Schematic model of Mib2-regulated-inflammation in microglia. Data indicate means ± SEM. Data were analyzed using one-way ANOVA. *p < 0.05, **p < 0.01, ***p < 0.001.

To study whether Mib2-mediated microglial activation is involved in this process, we performed Iba1 (a microglial marker) immunostaining and found the activated microglia in the ischemic penumbra at ipsilateral compared to corresponding contralateral position (Fig. 6A-6B). Further, the microglia in the ischemic penumbra in *Mib2* cKO mice showed robust branches and small soma compared with that of *Mib2^flox/flox^* mice (Fig. 6D). Besides, the number of microglia in *Mib2* cKO mice was reduced compared to the littermates of *Mib2^flox/flox^* mice (Fig. 6C). We also found that the inflammatory cytokines, including IL-6 and iNOS, were markedly decreased in the cKO mice brain (Fig. 6E-6H). Taken together, our results show that microglial deletion of *Mib2* mitigates ischemia-induced neuroinflammation and stroke-induced brain injury.

In summary, we demonstrate that Mib2 interacts with the IKK complex and promotes NF-κB signaling in the microglia. Furthermore, microglial deletion of *Mib2* reduces the microglial activation, neuroinflammation, and brain damage after ischemic stroke, suggesting that Mib2 might be a potential therapeutic target in stroke treatment (Fig. 6I).

## DISCUSSION

Microglial activation-induced neuroinflammation plays a critical role in many nervous system diseases including Alzheimer’s disease (AD), Parkinson’s disease (PD), and stroke [3-5, 37-40]. Mib2, an E3 ubiquitin ligase, has been reported to participate in regulating NF-κB signaling and type I IFN responses in the peripheral immune system. However, its role in microglia remained unknown. Here, we found that 1) Mib2 is involved in LPS- and OGD-induced microglial activation; 2) Mib2 interacts with IKK complex proteins and transcriptionally regulates Notch signaling through NF-κB activation; 3) Microglial specific *Mib2* knockout significantly alleviates microglial activation and ischemia-induced brain injury in mice, suggesting that Mib2 might be a potential therapeutic target.

Increasing evidence showed that the NF-κB pathway plays a critical role in ischemia-induced brain injury [32, 41]. Inhibition of NF-κB signaling using pharmacological inhibitors or knocking down p50 (an NF-κB subunit) in mice, reduced the brain injury and enhanced the functional recovery [32]. Our previous study showed that Hippo/MST signaling regulates the NF-κB pathway and mediate microglial activation in stroke [33]. Here, we found that Mib2 promotes NF-κB signaling in microglia by targeting the IKK complex, thus providing additional evidence for the detrimental role of NF-κB signaling in stroke.

It has been reported that Mib2 acts as an E3 ubiquitin ligase and post-translationally regulates Notch signaling. Using a prediction system, we found that most of the Mib2 substrates are associated with the Notch pathway. However, we found that knockdown of Mib2 decreased the levels of Notch1 and its activated form NICD in the microglia. Furthermore, we found that Mib2 promotes the activation of NF-κB signaling and transcriptionally regulates Notch expression, suggesting that Notch signaling could act as a downstream effector of the NF-κB pathway in LPS- and OGD-induced microglial activation. Also, we found that Notch signaling has a positive role in LPS- and OGD-induced microglial activation, which is consistent with previous studies [14, 42, 43]. Thus, we argue that NF-κB signaling could regulate Notch1 signaling and control the activation of microglia. Although we found that Mib2 interacts and ubiquitinates the IKK complex and regulates NF-κB signaling, the detailed regulatory mechanism needs further investigation. Future studies are required to address how Mib2 precisely regulates IKK complex.

It has been reported that *Mib2* knockout mice showed impairment in hippocampal spatial memory and contextual fear memory [20], indicating that Mib2 plays an important role in the central nervous system (CNS). However, the roles of Mib2 in the CNS pathophysiology are largely unknown. Since Mib2 is upregulated in animal models of tMCAo and plays an important role in the peripheral immune system, we speculated that Mib2 might play an important role in regulating microglial activation during stroke. To study the role of Mib2 in the microglia and to exclude the effect of peripheral immune cells, we generated microglial specific conditional *Mib2 *knockout mice, by crossing *Mib2^flox/flox^* mice with *Cx3cr1*^CreER^ mice [25, 44]. We are the first to report that microglial *Mib2* knockout significantly alleviates ischemia-induced brain injury, suggesting that microglial Mib2 has a detrimental role in this process. Microglial activation is always accompanied by neuroinflammation, an important target for therapeutic approaches to limit stroke damage [34, 35]. Here, we found that *Mib2* deficiency in microglia reduced microglial activation, including the number of microglia, the change of microglial morphology and the levels of related inflammatory cytokines.

In summary, our results suggest that Mib2 promotes microglial activation by regulating the NF-κB pathway. Microglial *Mib2* knockout protects ischemia-induced brain injury, suggesting that Mib2 might be a potential therapeutic target in stroke treatment.

## Supplementary Materials

The Supplemenantry data can be found online at: www.aginganddisease.org/EN/10.14336/AD.2019.0807.
