# Supplementary material for: Mib2 Deficiency Inhibits Microglial Activation and Alleviates Ischemia-Induced Brain Injury
Source: Aging Dis. 2020 Aug 7;11(3):523–35. doi: 10.14336/AD.2019.0807 (PMC7220279; doi:10.14336/AD.2019.0807)
Supplement: Supplementary file 1 — The Supplemenantry data can be found online at: www.aginganddisease.org/EN/10.14336/AD.2019.0807. [file AD-11-3-523-s.pdf]

SUPPLEMENTARY DATA

Mib2 Deficiency Inhibits Microglial Activation and Alleviates Ischemia-Induced Brain Injury

Xiaoheng Li[1], Yajin Liao[2,3], Yuan Dong[4], Shuoshuo Li[5], Fengchao Wang[6], Rong Wu[2], Zengqiang Yuan[1,2*], Jinbo Cheng[2,3*]

[1]Beijing Institute for Brain Disorders, Capital Medical University, Beijing, China
[2]The Brain Science Center, Beijing Institute of Basic Medical Sciences, Beijing, China
[3]Center on Translational Neuroscience, College of Life & Environmental Science, Minzu University of China, Beijing, China
[4]Department of Biochemistry, Medical College, Qingdao University, Qingdao, Shandong, China
[5]The State Key Laboratory of Brain and Cognitive Sciences, Institute of Biophysics, Chinese Academy of Sciences, Beijing, China
[6]National Institute of Biological Sciences, Beijing, China

© 2019. Li X et al. Published online at http://www.aginganddisease.org/EN/10.14336/AD.2019.0807

SUPPLEMENTARY DATA

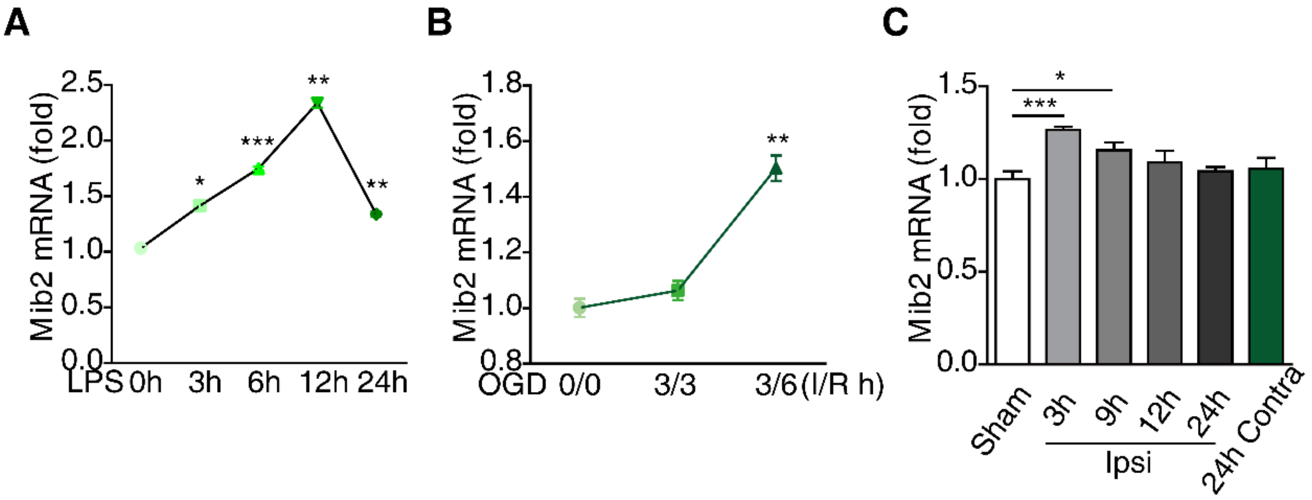

Supplementary Figure 1. **Mib2 is involved in the regulation of microglial mediated inflammation.** (**A**) RT-qPCR analysis of Mib2 expression levels in BV2 cells stimulated by LPS (1 µg/ml) for indicated times. (**B**) RT-qPCR analysis of Mib2 expression levels in BV2 cells upon oxygen-glucose deprivation treatment (OGD, ischemia for 3hr and reperfusion for indicated times), I: ischemia, R: reperfusion. (**C**) RT-qPCR analysis of Mib2 expression levels in brain tissues of mice from sham and MCAo groups for indicated reperfusion times. Data indicate means ± SEM. Data were analyzed using Student's *t* test. **p* < 0.05, ***p* < 0.01, ****p* < 0.001.

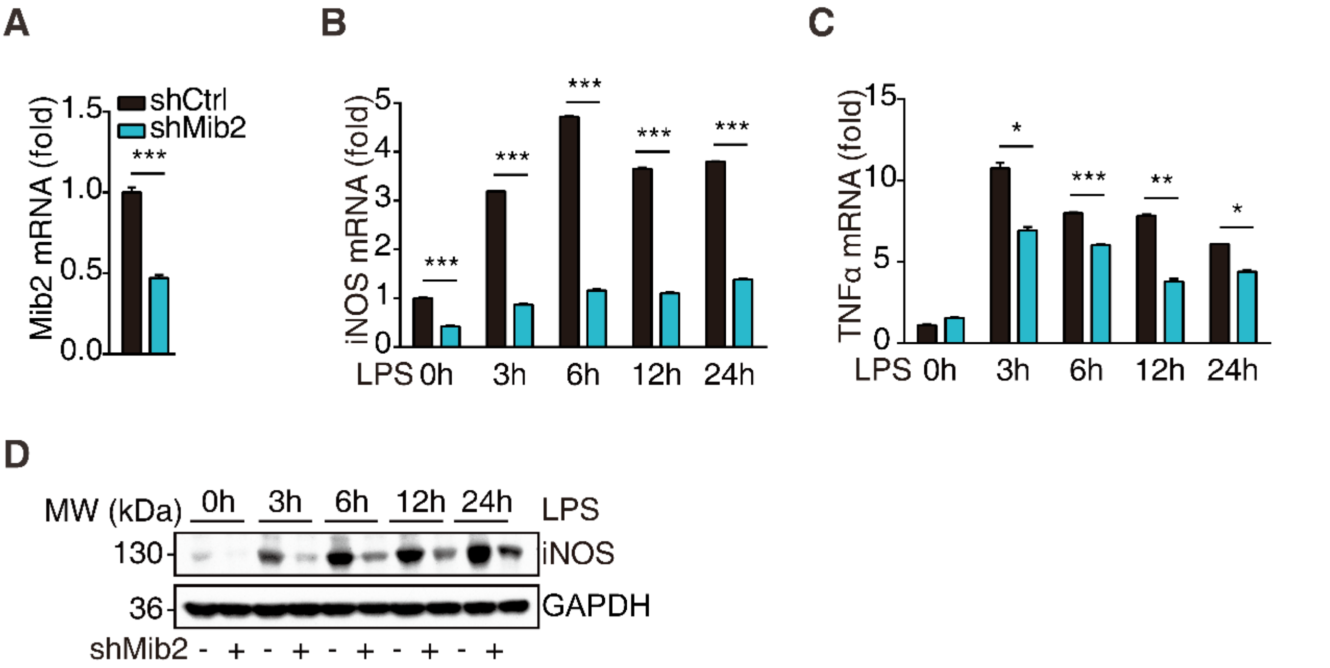

Supplementary Figure 2. Mib2 knockdown inhibits LPS-induced inflammation. (**A**) Mib2 knockdown efficiency of Mib2-knockdown stable BV2 cell line was determined by RT-qPCR analysis. (**B**) The expression levels of iNOS in control and Mib2 knockdown-BV2 cells were analyzed upon LPS (1 µg/ml) stimulation for indicated times. (**C**) The expression levels of TNFα in control and Mib2 knockdown-BV2 cells were analyzed upon LPS (1 µg/ml) stimulation for indicated times. (**D**) Western blot analysis of iNOS levels in control and Mib2 knockdown-BV2 cells upon LPS (1 µg/ml) stimulation for indicated times. Data indicate means ± SEM. Data were analyzed using one-way ANOVA. **p* < 0.05, ***p* < 0.01, ****p* < 0.001.

© 2019. Li X et al. Published online at http://www.aginganddisease.org/EN/10.14336/AD.2019.0807

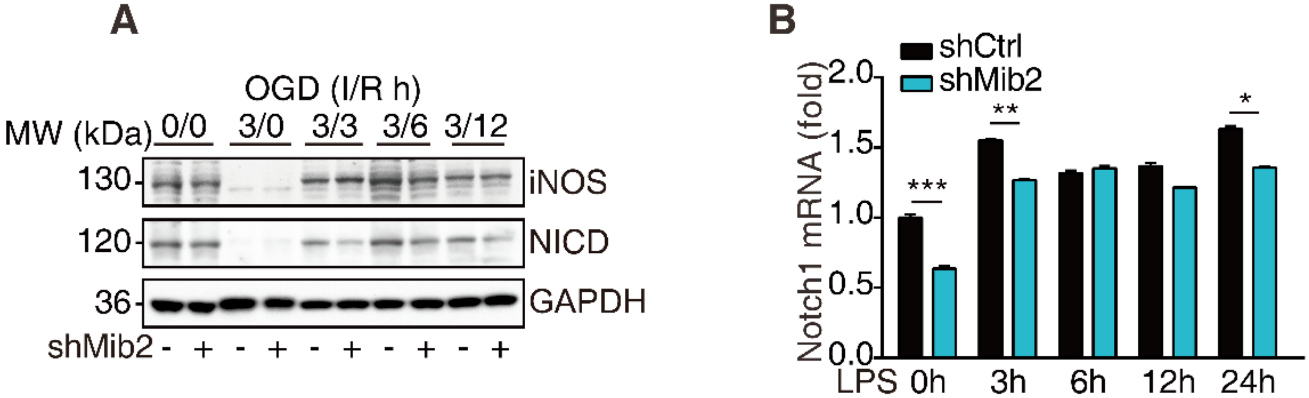

Supplementary Figure 3. Mib2 regulates Notch signaling pathway. (**A**) Western blot analysis of iNOS and NICD levels in control and Mib2 knockdown-BV2 cells upon OGD treatment (ischemia for 3hr and reperfusion for indicated times), I: ischemia, R: reperfusion. (**B**) The expression levels of Notch1 in control and Mib2 knockdown-BV2 cells were analyzed upon LPS (1 μg/ml) stimulation for indicated times. Data indicate means ± SEM. Data were analyzed using one-way ANOVA. **p* < 0.05, ***p* < 0.01, ****p* < 0.001.

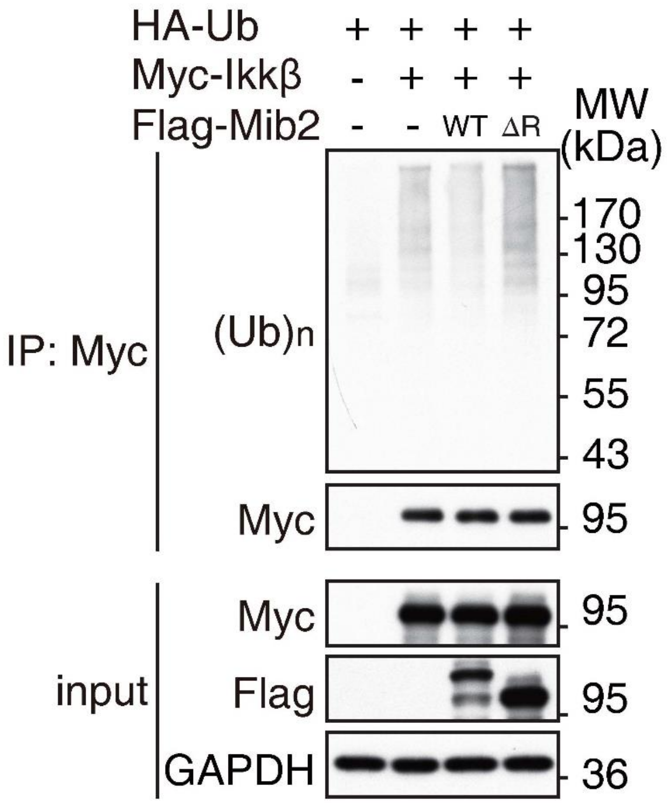

Supplementary Figure 4. Mib2 does not promote the ubiquitination of IKKβ. 293T cells were transfected with Flag-tagged Mib2 WT or ΔR (Ring domain deletion) or vector plasmid together with HA-tagged ubiquitin, Myc-tagged IKKβ. Cell lysates were immunoprecipitated with Myc antibody and immunoblotted with HA antibody.

© 2019. Li X et al. Published online at http://www.aginganddisease.org/EN/10.14336/AD.2019.0807

SUPPLEMENTARY DATA

Supplementary Table 1. The primers of the genes tested.

Target Gene	Primer type	Sequence
Mouse Mib2	Forward	5'-GGTTACGCTGAGCCCCCGACA-3'
	Reward	5'-ATCTTGTGAGCCCCATTCCCA-3'
Mouse INOS	Forward	5’-ATGGACCAGTATAAGGCAAGC-3’
	Reward	5’-GCTCTGGATGAGCCTATATTG-3’
Mouse IL-6	Forward	5'-GCTACCAAACTGGATATAATCAGGA-3'
	Reward	5'-CCAGGTAGCTATGGTACTCCAGAA-3'
Mouse TNF-α	Forward	5'-CAGGCGGTGCCTATGTCTC-3'
	Reward	5'-CGATCACCCCGAAGTTCAGTAG-3'
Mouse Notch1	Forward	5'-GATGGCCTCAATGGGTACAAG-3'
	Reward	5'-TCGTTGTTGTTGATGTCACAGT-3'
Mouse Hes1	Forward	5'-TCAGCGAGTGCATGAACGAG-3'
	Reward	5'-CATGGCGTTGATCTGGGTCA-3'
Mouse GAPDH	Forward	5’-AGGTCGGTGTGAACGGATTTG-3’
	Reward	5’-GGGGTCGTTGATGGCAACA-3’

© 2019. Li X et al. Published online at http://www.aginganddisease.org/EN/10.14336/AD.2019.0807
